# Outcome of Prostaglandin E1 Therapy in Critical Lower Limb Ischemia

**DOI:** 10.7759/cureus.102025

**Published:** 2026-01-21

**Authors:** Samaresh C Saha, M. D. Mezbahur Rahman, Mohammad A Islam, M. D. A Hossain, Tasnim A Khan, Muhammed I Khalilullah, Rajeebshankar Karmakar, Masnoon A Noor

**Affiliations:** 1 Vascular Surgery, Bangladesh Medical University, Dhaka, BGD; 2 Vascular Surgery, National Institute of Cardiovascular Diseases, Dhaka, BGD; 3 General Surgery, Directorate General of Health Services (DGHS), Dhaka, BGD; 4 Vascular Surgery, Social Islami Bank Limited (SIBL) Foundation Hospital, Dhaka, BGD; 5 Thoracic Surgery, Directorate General of Health Services (DGHS), Dhaka, BGD; 6 Thoracic Surgery, Bangladesh Medical University, Dhaka, BGD

**Keywords:** chronic critical limb ischemia, critical limb threatening ischemia, critical lower limb ischemia, peripheral arterial disease, prostaglandin, prostaglandin e1 therapy, prostaglandin therapy, rest pain, synthetic prostaglandin e1 analog, vascular claudication

## Abstract

Introduction: Critical lower limb ischemia (CLI) is a severe manifestation of peripheral arterial disease, often progressing to pain, tissue loss, and amputation when blood flow is critically compromised. As many patients are unsuitable for revascularization, prostaglandin E1 (PGE1) has emerged as a potential therapy to improve perfusion, relieve symptoms, and promote healing. This study evaluated the outcomes of intravenous PGE1 therapy at two major centers in Bangladesh, aiming to improve patients' quality of life.

Methodology: This observational study was conducted over a 12-month period in the vascular surgery departments of Bangladesh Medical University (BMU) and the National Institute of Cardiovascular Diseases (NICVD). The study population comprised patients with CLI receiving PGE1 therapy, selected through convenience sampling. Using a sample size calculation based on a prevalence rate of 7.5% for CLI, a total of 105 patients were included in the study. The inclusion criteria focused on patients aged 40 years or older with below-knee disease who were unsuitable for surgical intervention or had experienced failed revascularization. Exclusion criteria ruled out individuals with acute limb ischemia, vasculitis, aorto-iliac lesions, abdominal aortic aneurysms, or low cardiac function. All demographic and clinical information was documented in individual case record forms, and the compiled data were subsequently analyzed using appropriate statistical methods.

Results: The results of this study revealed important insights into the demographic and clinical characteristics of patients undergoing PGE1 therapy for CLI. Patients aged 70 years and above represented the largest group at 32.4%, with a predominance of male patients (90.5%) and a significant portion from lower socioeconomic backgrounds (76.2%). A substantial majority of patients were smokers (81%) and were nearly evenly split between diabetic and non-diabetic (48.6% and 51.4%, respectively). Hypertension and dyslipidemia were common comorbidities, affecting 57.1% and 47.6% of the cohort, respectively. A striking 93.3% of patients experienced rest pain, and 41.9% presented with ulceration. Despite the severity of the condition, PGE1 therapy demonstrated encouraging results, with 92.4% of patients reporting improvements in claudication, 81% experiencing relief from rest pain, and 56.2% showing ulcer healing. Only 5.7% of patients saw improvements in their ankle-brachial pressure index (ABPI). Around 71.4% of the cohort avoided amputation, and the in-hospital mortality rate was 8.4%, highlighting PGE1’s potential to improve patient outcomes in CLI.

Conclusions: PGE1 therapy showed meaningful clinical benefit in patients with CLI by relieving pain, improving claudication, promoting ulcer healing, and supporting limb salvage, even when objective perfusion gains were limited. These findings underscore its value as a practical therapeutic option in resource-limited settings and highlight the need for further studies to refine and optimize treatment strategies.

## Introduction

Critical lower limb ischemia (CLI), characterized by severely reduced blood flow due to arterial obstruction, poses a major threat of tissue loss, often demanding amputation [1]. CLI is manifested with chronic ischemic rest pain requiring analgesics or gangrene or non-healing ulcers, corresponding to Fontaine stage III and IV peripheral arterial disease [2]. Even with revascularization attempts, the one-year mortality rate exceeds 50% in CLI patients, with 75% undergoing amputation or experiencing persistent pain or ulcers [1]. For patients unsuitable for revascularization, medical therapy with prostaglandin E1 (PGE1) has emerged as an effective option to relieve symptoms and achieve limb salvage. 

PGE1 improves limb perfusion in CLI by its vasodilatory and platelet aggregation inhibitory action [3,4]. PGE1 does not reduce systemic arterial pressure due to a compensatory cardiac output rise [4]. Proposed CLI treatment mechanisms include inhibiting platelet activation/aggregation, improving endothelial function, reducing blood viscosity, stimulating angiogenesis, and enhancing microvascular flow [4,5]. By providing symptomatic relief in CLI and avoiding major amputation, PGE1 therapy can preserve quality of life and functional status in patients having an otherwise poor prognosis [1]. Based on early evidence, PGE1 gained recommendation for managing no-option CLI patients to relieve rest pain, heal ulcers, prevent disability, and prolong viability [2]. Meta-analyses have found it to be the most effective and well-tolerated prostaglandin for CLI treatment [2]. A multicenter, placebo-controlled randomized trial in patients unsuitable for revascularization showed intravenous PGE1 led to significant reductions in rest pain versus placebo and strong but non-significant trends toward ulcer healing and limb salvage [6]. This trial demonstrated clear patient benefit from PGE1, confirming its use for CLI, but left questions regarding optimal protocols.

Numerous randomized controlled trials originating predominantly in Japan have supported PGE1 efficacy in CLI while helping optimize its clinical application [5]. A substantial body of literature now supports incorporating PGE1 into CLI management, especially for patients ineligible for mechanical revascularization. PGE1 provides symptom relief while reducing rates of disability and death in this population through enhancing limb perfusion [5,6]. Despite over 40 years of accumulating evidence, important clinical questions persist regarding PGE1 therapy for CLI. While studies have demonstrated treatment efficacy, optimal protocols for dosing, route, and duration remain unclear. Data directly comparing PGE1 to other medical options are lacking. Moreover, nearly all randomized trials have been conducted internationally, not originating from Bangladesh, raising uncertainty about outcomes within the country’s healthcare setting and patient demographics.

This study aimed to investigate the effects of intravenous PGE1 therapy for CLI at Bangladesh Medical University (BMU) and the National Institute of Cardiovascular Diseases (NICVD) in Dhaka, Bangladesh, with a focus on patients who could not undergo revascularization. As leading centers, BMU and NICVD were selected due to their high patient volumes and advanced clinical capabilities, making them ideal settings for evaluating CLI treatment outcomes. The study assessed the impact of PGE1 therapy on pain relief, ulcer healing, limb preservation, and survival rates among patients with CLI.

## Materials and methods

Study design and procedure

This prospective observational study was conducted between July 2023 and August 2024 in the vascular surgery departments of BMU and NICVD. Using a convenience sampling technique, 105 patients with CLI who were receiving PGE1 therapy were enrolled. Patients aged 40 years or older with below-the-knee disease who were unsuitable for endovascular or open surgical intervention or who had failed prior interventions were included, while those with acute limb ischemia, vasculitis, aorto-iliac involvement, abdominal aortic aneurysm, or compromised cardiac function were excluded. Following ethical approval from the institutional review board (IRB) of BMU and the acquisition of written informed consent, diagnosis was established through clinical evaluation, Doppler study, and CT angiogram or peripheral angiogram (PAG), with symptom severity assessed using Fontaine’s grading system and clinical parameters including severe claudication, rest pain, ulceration, and amputation status [7].

Injection PGE1 was administered as a continuous slow intravenous infusion once a day for three days a month (one course) for up to six months. Each ampoule contained 500 micrograms of PGE1, which was diluted with 8 mL of normal saline in a 10-mL syringe. A total of 3 mL (equivalent to 100 micrograms) was placed in 500 mL of normal saline and given as a continuous intravenous infusion with a microdrip set (Yash Care Lifesciences, Ahmedabad, India). This was administered slowly (over 30 minutes to hours, depending on the patient's condition) because rapid infusion could induce myocardial ischemia due to a coronary steal effect produced by peripheral vasodilatation. After completion of therapy, patients were followed up every month up to six months for evaluating longer-term outcomes such as ulcer healing, improvement in peripheral pulsations, and changes in amputation level, with all assessments performed through clinical examination and Doppler study.

Data recording

All demographic and clinical details of the study participants were recorded in individual case record forms (see Appendix A-C).

Statistical analysis

Collected data were carefully checked, edited, and encoded before being entered into IBM SPSS Statistics version 22.0 (IBM Corp., Armonk, USA) and Microsoft Excel (Microsoft Corp., Redmond, USA) for statistical analysis. Quantitative variables were expressed as mean ± standard deviation (SD) and compared using the Student’s t-test for two groups. Qualitative or categorical variables were presented as frequencies and proportions, with comparisons made using the chi-square test when appropriate. All analyses were performed at a 5% level of significance (p<0.05).

Ethical considerations

Ethical clearance for the study was obtained from the IRB of BMU, and necessary permissions were also secured from the relevant departments of the institute. In adherence to the Helsinki Declaration of 2011 concerning medical research involving human subjects, all participants were thoroughly informed about the study design and objectives prior to their inclusion. They were explicitly assured of their right to withdraw from the study at any point and for any reason, without any consequence. Written informed consent was obtained from each participant through a transparent and respectful process that clearly outlined the potential benefits and risks associated with the procedure. Confidentiality was strictly maintained, and no data were disclosed without the explicit permission of the respondents. No force was applied, and interviews were conducted only with those who willingly agreed to participate.

## Results

Table 1 portrays a cohort largely composed of older adults (61.7 ± 12.3 years), with most patients falling into the later decades of life and a modest male predominance. The majority belonged to a lower socioeconomic background, and lifestyle-related and metabolic comorbidities were frequent, including a high proportion of smokers alongside substantial burdens of diabetes, hypertension, and dyslipidemia. Table 2 illustrates that rest pain was the most prevalent presenting symptom, showing the severity of ischemic compromise in this cohort. Ulceration and gangrene were also common, reflecting advanced tissue involvement and highlighting the chronicity and progression of ischemic disease among the respondents. Table 3 demonstrates that the majority of patients had markedly reduced ankle-brachial pressure index (ABPI) values (0.40-0.59), with most clustering within the moderate-to-severe ischemic range [8]. The absence of patients at the extremes of the ABPI scale suggests that the cohort was predominantly characterized by substantial, but not end-stage, hemodynamic impairment, aligning with the clinical diagnosis of CLI [1]. Table 4 reveals a varied distribution in the number of therapy courses received, with a notable proportion completing the full treatment regimen while others discontinued earlier. This pattern suggests differences in clinical response, tolerance, or follow-up adherence, offering insight into the practical trajectory of PGE1 therapy in real-world clinical settings.

**Table 1:** Distribution of respondents according to demographic variables

Variables	Frequency	Percentage
Age (years)		
40-49	22	21
50-59	20	19
60-69	29	27.6
≥70	34	32.4
Sex		
Male	95	90.5
Female	10	9.5
Socioeconomic status		
Upper	10	9.5
Middle	15	14.3
Lower	80	76.2
Smoker	85	81
Diabetic	51	48.6
Hypertensive	60	57.1
Dyslipidemia	50	47.6

**Table 2:** Distribution of respondents according to clinical symptoms

Symptoms	Frequency	Percentage
Rest pain	98	93.3
Ulceration	44	41.9
Gangrene	27	25.7

**Table 3:** Distribution of respondents according to ABPI ABPI: ankle-brachial pressure index

ABPI level [8]	Frequency	Percentage
<0.40	0	0
0.40-0.59	72	68.6
0.60-0.79	33	31.4
0.80-0.90	0	0

**Table 4:** Distribution of respondents according to number of therapy

Number of therapy	Frequency	Percentage
6 courses	44	41.9
5 courses	9	8.6
4 courses	12	11.4
3 courses	10	9.5
2 courses	10	9.5
1 course	20	19.1

Table 5 reflects a broadly favorable clinical response to therapy, as most patients experienced notable improvement across key ischemic symptoms. Claudication showed the highest degree of recovery, followed by substantial relief of rest pain, while a meaningful proportion also demonstrated progress in ulcer healing. Table 6 reveals that improvement in ABPI was observed in only a small fraction of patients, indicating that hemodynamic enhancement was limited despite the symptomatic gains noted earlier [8]. This disparity between clinical relief and objective vascular measurement suggests that PGE1 may offer symptomatic benefit without producing substantial changes in measurable limb perfusion. Table 7 highlights the gravity of disease severity within the study population, as a considerable proportion ultimately required amputation and a large share experienced in-hospital mortality. This table shows the critical and life-threatening nature of advanced lower limb ischemia, illustrating that despite therapeutic efforts, outcomes remained poor for many individuals.

**Table 5:** Distribution of respondents according to improvement in clinical symptoms

Improvement in clinical symptoms	Frequency	Percentage
Claudication	97	92.4
Rest pain	85	81
Ulcer healing	59	56.2

**Table 6:** Distribution of respondents according to improvement in ABPI ABPI: ankle-brachial pressure index

Improvement in ABPI [8]	Frequency	Percentage
Improved	6	5.7
Not improved	99	94.3

**Table 7:** Distribution of respondents according to need for amputation and in-hospital mortality

Amputation	Frequency	Percentage
Amputation done	30	28.6
In-hospital mortality	9	8.4

## Discussion

CLI represents the most advanced stage of peripheral arterial disease, often leading to debilitating pain, tissue loss, and a high risk of limb loss and mortality. PGE1 therapy has emerged as a potential limb-saving intervention, offering vasodilatory and microcirculatory benefits. In our study, the patients were predominantly older adults with a slight male predominance, most belonging to a lower socioeconomic background and carrying multiple vascular risk factors such as smoking, diabetes, hypertension, and dyslipidemia, an overall profile consistent with advanced peripheral arterial disease. Comparable age distributions have been documented in several studies, with one reporting a common age range of 60-70 years, another noting a mean age of 71.2 ± 10.7 years, and a further study with a similar finding of 72.5 ± 10.7 years [1,9,10]. The male predominance in our cohort is also aligned with earlier findings, with 67% male participation reported in one study, 69.8% male distribution observed in another, and approximately 50-51% male proportions recorded in other large samples [1,6,9,10]. The association between low socioeconomic status and CLI is similarly supported by prior work, with one study showing that lower socioeconomic status significantly increased the odds of presenting with chronic limb-threatening ischemia [11]. The high prevalence of smoking in our population reflects established epidemiological patterns, as earlier studies documented smoking rates of 50% and 21.3% among similar patient groups [10,12]. Hypertension was also frequent in our cohort, consistent with reports showing 23.8% prevalence in one study and markedly higher rates of 86.9% in another [5,10]. Additionally, the burden of diabetes in our sample mirrors findings from previous work, with reported rates of 39.7%, 50%, 55.2%, and 65.6% [5,9,10,12]. Dyslipidemia, also common in our population, corresponds to the 44.3% prevalence noted in another study [10]. 

Most patients presented with severe ischemic symptoms, with rest pain being particularly predominant, while ulceration and gangrene were also frequent, reflecting the advanced stage of disease. This pattern resonates with findings from other research, where one study showed rest pain in 95.1% of cases, whereas another reported a lower prevalence of 48.88% [1,10]. Ulceration in our cohort appeared moderately common, situating itself between the higher rate of 81.9% reported in one study and the lower rate of 31.11% observed elsewhere [1,10]. Gangrene, while present in a subset of our patients, was reported at much higher levels in other investigations, with 80% documented in one study and 80.6% in another, highlighting variability across clinical settings [1,9]. The ABPI values in our population reflected moderate to severe ischemia, closely aligning with previously published measurements, such as 0.64 in the right limb and 0.57 in the left limb in one series, 0.60 ± 0.04 in another, and 0.65 ± 0.2 in a third [7,9,13,14]. The distribution of therapy courses in our study demonstrated a pattern remarkably similar to earlier reports, where one study showed 44.4% of patients completing six courses, with gradually fewer completing five, four, three, or two courses, and 20% receiving a single course [1]. These comparisons reveal that while our cohort reflects the typical clinical severity seen in critical limb ischemia, notable variations across studies underscore the heterogeneity inherent in this high-risk patient population.

Most patients experienced meaningful improvement in claudication and rest pain, and more than half showed progress in ulcer healing, illustrating a generally favorable symptomatic response to PGE1 therapy. Objective hemodynamic improvement was rare, and a substantial proportion ultimately required amputation or succumbed during hospitalization, reflecting the advanced severity of their disease. These findings resonate with prior literature. One study showed 91% improvement in claudication distance, while another reported a 123% increase in walking distance [6,15]. Improvement in rest pain varied widely across studies, ranging from 0% in one report to 86.2% in another [10,12]. Ulcer healing outcomes also differed, with one study reporting 0% improvement, whereas others documented healing in 41% of cases and 58% improvement in a separate cohort [10,12,16]. Similar to our population, amputation remained a significant outcome in the literature, reported as 34.3% in one study, 22.9% in another, and reaching 46.6% in an earlier series [4,9,10,17]. Consistent with our limited ABPI improvement, several studies observed 0% change in perfusion indices [7,12,13]. In-hospital mortality rates across studies also varied widely, from 40.3% to 9.8%, with one earlier report noting 12% mortality [4,9,10]. These comparisons suggest that while symptomatic relief with PGE1 is frequently observed, objective vascular improvement and limb salvage remain challenging, particularly in patients presenting at an advanced stage of CLI.

## Conclusions

In this study, PGE1 therapy was associated with clinically meaningful improvement in symptoms among patients with CLI, particularly with respect to pain relief and functional comfort. While these findings suggest a potential beneficial role for PGE1 in patients with limited therapeutic options, they should be interpreted with caution. The observational design, short follow-up duration, and absence of a comparator group preclude definitive conclusions regarding causality or long-term benefit. Although efforts were made to reduce selection and information bias through predefined eligibility criteria, consecutive enrollment, standardized treatment protocols, and prospective data collection, variability in comorbid conditions and reliance on largely subjective outcome measures may have influenced the observed responses. The tertiary-care setting and convenience sampling limit generalizability. These results support an association between PGE1 therapy and symptomatic improvement but highlight the need for more rigorous controlled studies with larger and well-defined populations to establish its efficacy, durability, and role as a definitive therapeutic modality in CLI.

## Appendices

Appendix A 

**Table 8:** Pre-therapy details This data collection form was created by the authors. BMU: Bangladesh Medical University; NICVD: National Institute of Cardiovascular Diseases

Item	Details/options
Administrative	
Case no.	__________________________
Name of the institute	☐ BMU ☐ NICVD
Ward	__________________________
Bed	__________________________
Hospital registration no.	__________________________
Date of admission	__________________________
Date of data collection	__________________________
Date of prostaglandin E1 therapy starting	__________________________
Patient’s details	
Name	__________________________
Age	__________________________
Sex	__________________________
Address	__________________________
Phone no.	__________________________
Monthly income	__________________________
Risk factors	
History of smoking	☐ Present ☐ Absent
History of hypertension	☐ Present ☐ Absent
History of diabetes mellitus	☐ Present ☐ Absent
History of dyslipidemia	☐ Present ☐ Absent
History of cardiovascular disease	☐ Present ☐ Absent
Pre-therapy evaluation	
Presence of severe claudication (Fontaine IIB)	☐ Yes ☐ No
Presence of rest pain	☐ Yes ☐ No
Presence of ulceration	☐ Yes ☐ No
Presence of gangrene	☐ Yes ☐ No
History of previous intervention	☐ Yes ☐ No
Pre-therapy ankle-brachial pressure index (ABPI) [8]	Value: __________________________
Number of prostaglandin E1 therapy/cycle	
Course 1	☐
Course 2	☐
Course 3	☐
Course 4	☐
Course 5	☐
Course 6	☐

Appendix B

**Table 9:** Post-therapy evaluation This data collection form was created by the authors.

Assessment item	1st visit	2nd visit	3rd visit	4th visit	5th visit	6th visit
Post-therapy claudication distance	☐ Improved ☐ Not improved	☐ Improved ☐ Not improved	☐ Improved ☐ Not improved	☐ Improved ☐ Not improved	☐ Improved ☐ Not improved	☐ Improved ☐ Not improved
Post-therapy rest pain	☐ Improved ☐ Not improved	☐ Improved ☐ Not improved	☐ Improved ☐ Not improved	☐ Improved ☐ Not improved	☐ Improved ☐ Not improved	☐ Improved ☐ Not improved
Post-therapy ulcer healing	☐ Improved ☐ Not improved	☐ Improved ☐ Not improved	☐ Improved ☐ Not improved	☐ Improved ☐ Not improved	☐ Improved ☐ Not improved	☐ Improved ☐ Not improved
Post-therapy need for amputation	☐ Needed ☐ Not needed	☐ Needed ☐ Not needed	☐ Needed ☐ Not needed	☐ Needed ☐ Not needed	☐ Needed ☐ Not needed	☐ Needed ☐ Not needed
Post-therapy ankle-brachial pressure index (ABPI) [8]	Value (1st): ______	Value (2nd): ______	Value (3rd): ______	Value (4th): ______	Value (5th): ______	Value (6th): ______

Appendix C

**Table 10:** Mortality status This data collection form was created by the authors.

Mortality	Yes	No
Status	☐	☐
